# Liquid–Liquid Interface Polar Engineering for Organic Piezoelectrics

**DOI:** 10.34133/research.1003

**Published:** 2025-11-25

**Authors:** Yongkang Zhang, Shuhai Liu

**Affiliations:** School of Materials and Energy, Lanzhou University, Lanzhou, China.

## Abstract

Organic piezoelectric materials have garnered increasing interest for biomechanical applications; however, their practical appeal has been limited by low piezoelectricity and low mechanical compliance. Recently, liquid–liquid interface polar engineering has emerged as a promising strategy to overcome these limitations. By utilizing polarity asymmetry at the liquid–liquid interface, this strategy enables precise control over molecular assembly and phase separation, thereby inducing high piezoelectric polarization of organic materials while maintaining their intrinsic mechanical compliance. This breakthrough paves the way for developing organic piezoelectric materials that exhibit both high piezoelectricity and high mechanical compliance, advancing their potential in biomechanical sensing, actuating, and energy harvesting.

Organic piezoelectric materials are gaining growing attention in biomechanical sensing, actuation, and energy harvesting. These applications require materials that combine high piezoelectricity with high mechanical compliance to enable high-fidelity detection of physiological signals [1]. High piezoelectricity reflects the efficiency of converting subtle mechanical stimuli into electrical signals, while high mechanical compliance requires the material with a low Young’s modulus to be well matched with biological tissues, preventing stress concentration or shielding effects. Nevertheless, simultaneously fulfilling both criteria remains a major challenge for organic piezoelectric materials [2]. Their piezoelectric coefficients (typically *d*_33_ < 30 pC/N) are substantially lower than those of inorganic piezoelectric materials such as lead zirconium titanate, which can exhibit a *d*_33_ of up to 200 pC/N. This inherent limitation compromises the signal-to-noise ratio in detecting faint physiological activities such as pulse or muscle microvibration. Moreover, the Young’s modulus of most organic piezoelectric materials (on the order of gigapascals) exceeds that of biological tissues (kilopascals to megapascals) by 3 to 6 orders of magnitude. Such a mismatch not only impedes signal transmission efficiency but also raises the risk of inflammatory responses in surrounding tissues.

These limitations stem from a fundamental contradiction between piezoelectricity and mechanical compliance caused by the material’s microscopic structure [3]. High piezoelectricity requires a highly ordered polar crystalline structure to enable efficient mechanical-to-electrical conversion. However, such structural order increases spatial steric hindrance, restricting the internal rotation and slippage of molecular chains and thereby reducing mechanical compliance. Conversely, achieving rubberlike mechanical compliance necessitates greater freedom and weaker intermolecular interaction, which inevitably promotes structural disorder and thus diminish piezoelectricity. This “order–disorder” dichotomy poses a central challenge in molecular design: strengthening intermolecular interactions and polar molecular chain alignment can enhance piezoelectricity but concurrently impedes molecular chain mobility. Conventional modification strategies—such as blending or plasticizing [4]—often improve one property at the expense of the other. For instance, introducing fillers can increase the dielectric constant and thus enhance piezoelectricity but compromises mechanical compliance; supramolecular cross-linking may increase mechanical compliance but reduce piezoelectricity. It is difficult to break through the intrinsic conflict between piezoelectricity and mechanical compliance. Therefore, innovative design concepts are urgently needed to overcome this inherent conflict and guide the development of high-performance organic piezoelectric materials.

Liquid–liquid interface polar engineering has emerged as a promising strategy to address these challenges. This strategy achieves efficiency and order by regulating the interface polarity gradient, tension, and molecular amphiphilicity. This process relies on 3 key conditions: interface potential difference provided by the polar/nonpolar solvent pair, solute molecules containing oriented functional groups (–OH and –NH_2_), and a controllable self-assembly driving force such as solubility differences or solvent evaporation rates. In such a liquid-phase system, the molecular chains approach a free state, with markedly weakened interchain interactions and steric hindrance, thereby achieving higher mobility freedom. When a polarity-gradient-induced polarized field forms at the solvent–solvent interface, polar groups within the molecular chains experience an asymmetric force that drive their alignment along the direction of polarized field (Fig. 1A). This property makes the molecular chains more susceptible to the influence of the polarized field at the liquid–liquid interface, enabling directed self-assembly (Fig. 1A (i)) and phase separation (Fig. 1A (ii)) at the molecular level through the Marangoni effect and interfacial energy differentials, thereby creating a pronounced asymmetry structure across the interface. It is worth pointing out that by introducing amphiphilic polymer composites, nonpolar molecules can also participate in this assembly process. This mechanism offers a new pathway for designing organic materials with high piezoelectric performance. Several studies have demonstrated the effectiveness of this method.

**Fig. 1.**
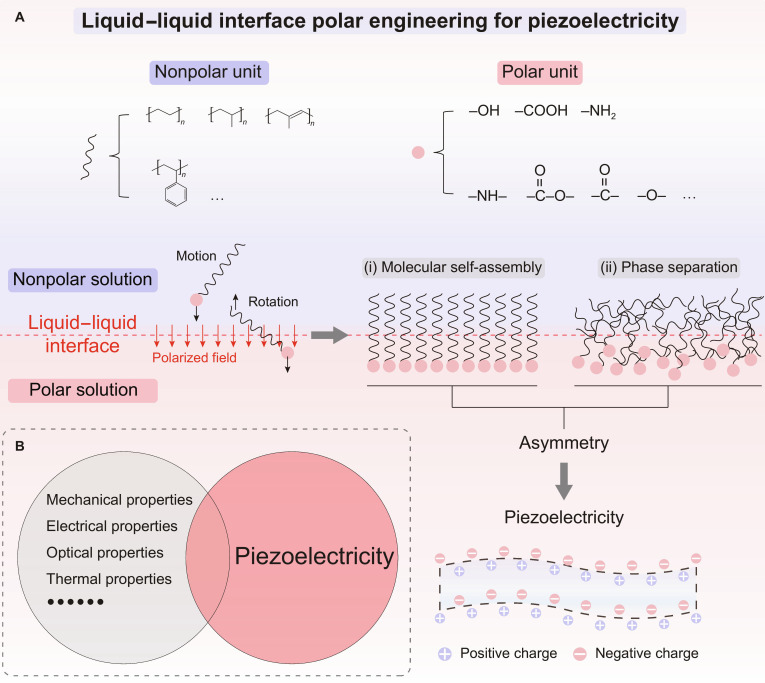
(A) Liquid-liquid interface polar engineering for piezoelectricity. Two kinds of interface assembly structures: (i) molecular self-assembly; (ii) phase separation. (B) Prospects for the coupling of piezoelectricity and multi-physical properties in organic materials.

Based on this strategy, a wafer-scale γ-glycine bio-piezoelectric film by leveraging solute solubility gradients was developed by Xudong Wang’s team in 2021 [5]. During solvent evaporation, polyvinyl alcohol (PVA) preferentially segregated at the air–liquid interface. Under the interfacial polarized field, the hydroxyl groups on PVA chains underwent solvent-oriented alignment and bonded with the carboxyl oxygen atoms of glycine molecules, thereby inducing γ-phase directional crystallization. Recently, a chiral noncentrosymmetric 1-hydroxy naphthaldehyde-(*S*)-3-aminopropane-1,2-diol organic single crystal was successfully synthesized by utilizing the limited miscibility and density differences of a hexane and dichloromethane/methanol mixed solvent in 2025 [6]. The hydrogen bonds and C–H···π interactions driven by the 1-hydroxy naphthaldehyde unit, in conjunction with the enhanced hydrogen-bonding network formed by the imine and hydroxyl groups, facilitate the directional stacking of molecules, thereby exhibiting a noncentrosymmetric structure. These represent important progress in introducing piezoelectricity by self-assembly via interface polar engineering (Fig. 1A (i)). Note that these piezoelectrics constructed by self-assembly are constrained by their requirement for a high degree of crystalline order. Meanwhile, Ren-Gen Xiong’s team [7] achieved high piezoelectricity in HOCH_2_(CF_2_)_3_CH_2_OH [2,2,3,3,4,4-hexafluoropentane-1,5-diol (HFPD)] by utilizing a 2-phase solvent system to form a 2-dimensional hydrogen-bonding network via O–H···O interactions of its terminal hydroxyl groups, combined with the orderly arrangement of fluorine atoms. They fabricated a highly flexible piezoelectric composite film by compositing HFPD with PVA, opening new possibilities for biomedical applications.

More recently, Zhang et al. [2] employed a water–oil interface to dissolve the linear polymers polyethylene glycol (PEG) and polystyrene-*block*-polyisoprene-*block*-polystyrene (SIS), which produces a strongly polarized field at the interface, driving phase separation of PEG and SIS to form an asymmetric structure (Fig. 1A (ii)). The intrinsically low steric hindrance of the linear polymer backbone, together with the formed asymmetric structure, endows the material with both high mechanical compliance and high piezoelectricity. Based solely on polarity asymmetry rather than crystallinity, this strategy breaks through the limitations of material systems. In theory, it can introduce piezoelectricity into almost any organic material containing polar functional groups, including amorphous polymers, elastomers, and biopolymers, which usually cannot achieve appreciable piezoelectricity through electric polarization or chemical modification methods. Furthermore, by selecting or designing materials with specific functional polar groups (such as photosensitive groups, thermoresponsive groups, and bioactive groups), the piezoelectric effect can be actively integrated with other physical or chemical properties (like photoresponse, thermoresponse, and biosensing) into a single material system. This approach not only greatly expands material selectivity but also offers substantial potential for integrating piezoelectricity with multi-physical properties (Fig. 1B).

Liquid–liquid interface polar engineering meets the “dual-high” performance of piezoelectricity and mechanical compliance for advanced organic piezoelectrics. It is expected to become one of the core strategies for the preparation of high-performance organic piezoelectric materials. By designing novel polar molecules, this strategy is expected to substantially expand the material selection and multifunctionality in the fields of biomechanical sensing, actuation, energy harvesting, and beyond in the future, offering continuous impetus for innovation in these rapidly evolving fields. However, several challenges remain in practical applications. For instance, temperature fluctuations can alter interfacial tension and molecular thermal motion, thereby affecting structural stability. Meanwhile, changes in humidity may cause swelling or even degradation of moisture-sensitive components, disrupting interfacial asymmetry. To overcome these challenges, future efforts should focus on optimizing material systems (e.g., hydrophobicity and glass transition temperature) and interfacial structures (e.g., introducing cross-linking and hydrophobic coatings), or employing encapsulation strategies, to enhance their robustness under complex environmental conditions.
